# The protective effect of L-carnitine on testosterone synthesis pathway, and spermatogenesis in monosodium glutamate-induced rats

**DOI:** 10.1186/s12906-022-03749-0

**Published:** 2022-10-13

**Authors:** Farhad Koohpeyma, Fatemeh Gholizadeh, Hannaneh Hafezi, Mehri Hajiaghayi, Morvarid Siri, Shaghayegh Allahyari, Mohammad Hasan Maleki, Naeimehossadat Asmarian, Elahe Bayat, Sanaz Dastghaib

**Affiliations:** 1 Endocrinology and Metabolism Research Center, Shiraz University of Medical Sciences, P.O. Box: 71345-1744, Shiraz, Iran; 2 Department of Biology, Concordia University, Montreal, QC Canada; 3 Autophagy Research Center, Shiraz University of Medical Sciences, Shiraz, Iran; 4 Department of Genetics, Arsanjan Branch, Islamic Azad University, Fars, Iran; 5 Department of Biochemistry, School of Medicine, Shiraz University of Medical Sciences, Shiraz, Iran; 6 Anesthesiology and Critical Care Research Center, Shiraz University of Medical Sciences, Shiraz, Iran

**Keywords:** Male infertility, MSG, L-carnitine, Anti-oxidant, Testosterone synthesis, Spermatogenesis

## Abstract

**Background:**

Monosodium glutamate (MSG) is a food ingredient that is increasingly used commercially. MSG leads to oxidative stress, consequently suppressing steroid hormone production that causes defects in male reproductive system. This study aimed to evaluate the effect of L-carnitine as an antioxidant on testicular damage in MSG-induced male rats.

**Methods:**

Sixty adult male Spargue-Dawley rats were randomly divided into six groups of ten as follows: control (water), sham (normal saline), L-carnitine (200 mg/kg b.w), MSG (3 g/kg b.w), MSG + L-carnitine 100 (3 g/kg b.w of MSG and 100 mg/kg b.w of L-carnitine), and MSG + L-carnitine 200 (3 g/kg b.w of MSG and 200 mg/kg b.w of L-carnitine). The treatment was administered by oral gavage for six months. Serum levels of Malondialdehyde (MDA), Total Anti-oxidant Capacity (TAC), LH, FSH, testosterone, and mRNA expressions of *Star, Cyp11a1, and Hsd17b3* genes, and histological and stereological changes were assessed.

**Results:**

L-carnitine led to a significant decrease in the level of MDA and a significant rise in the serum levels of TAC, LH, FSH, and mRNA expression of *Star* and *Cyp11a1* compared to the MSG group (*p* < 0.05)*.* Furthermore, stereological results indicated a significant increment in the number of sexual lineage cells, the total volume of the testis, length, diameter, and volume of seminiferous tubules, the height of the germinal epithelium, sperm count, and sperm motility (*p* < 0.05) in MSG + L-carnitine 200 compare to MSG group.

**Conclusion:**

The study’s findings demonstrated that L-carnitine due to its anti-oxidant properties, ameliorated the reproductive abnormalities in the male rats exposed to MSG.

## Background

Infertility refers to the inability to become pregnant after 12 months of regular intercourse without the use of contraceptives. It has been estimated that 8–12% of couples in reproductive ages are infertile, with half of the cases being attributed to male factors [1]. Smoking, alcohol, lifestyle, and food can contribute to male reproductive disorders [2]. Monosodium Glutamate (MSG) is a food additive used to enhance the flavor and food taste [3]. A systematic review reported that MSG led to oxidative stress, thereby causing male reproductive toxicity through a variety of pathways including oxidative damage, histomorphological changes, hormone dysfunction, and sperm quality reduction [4].

Oxidative stress has negative impacts on steroidogenic enzymes through suppressing steroid hormone production [5]. The hypothalamic-pituitary–gonadal axis regulates testosterone levels and gonadotropin production through stimulating anterior pituitary and secretion of Luteinizing Hormone (LH) and Follicle-Stimulating Hormone (FSH). Sertoli cells are stimulated by FSH to promote sperm production and regulate spermatogenesis [6]. LH regulates Leydig cell activity and immensely controls testicular function. Leydig cells generate and release testosterone as a key male sex hormone that is essential for male growth and reproduction [7]. Two key proteins involved in the initial phases of steroidogenesis are Steroidogenic Acute Regulatory protein (*Star*) and cytochrome P450 family 11 subfamily a polypeptide 1 (*Cyp11a1*) [7]. 17β-Hydroxysteroid dehydrogenase-3 (*Hsd17b3*) is also one of the most influential enzymes in the conversion of androstenedione to testosterone in Leydig cells [8]. Many infertile males have low testosterone levels [9], which can be due to a decrease in the activities of *Star*, *Cyp11a1*, and *Hsd17b3* in Leydig cells [10].

Infertile males can benefit from a wide variety of hormonal treatments and antioxidant supplementation aimed at increasing endogenous FSH, androgen levels, and spermatogenesis [11, 12]. Using L-carnitine (LC) as a potent antioxidant, on the other hand, can be a sensible and effective method of treating male infertility [13]. LC is a physiologically active amino acid found in meat and milk, which are the most common dietary sources of exogenous carnitine for humans [14]. LC has anti-inflammatory, anti-apoptotic, neuroprotective, cardioprotective, and gastroprotective properties due to its antioxidant and free radical scavenging properties [13]. Moreover, owing to its antioxidant properties, LC protects the body from Reactive Oxygen Species (ROS) detrimental effects. ROS can damage unsaturated fatty acids and diminish the sperm plasma membrane fusogenicity, while LC preserves spermatozoa from oxidative stress through removing excess intracellular toxic acetyl coenzyme A and replacing fatty acids in membrane phospholipids [15]. Several investigations have demonstrated the positive therapeutic impacts of LC on infertile males. Seminal carnitine content has been found to be associated with sperm count and motility [15–19]. However, the exact therapeutic effect of LC on testicular steroidogenesis, spermatogenesis, stereological characteristics, and reproductive function has remained uncertain.

Given that the consumption of MSG, a flavor enhancer, has increased and some findings have proved its deleterious effects on the reproductive system of male rats [20–23], the present study aimed to evaluate the potential therapeutic effect of LC on LH, FSH, and testosterone levels, mRNA expression levels of *Star*, *Cyp11a1*, and *Hsd17b3*, and stereological parameters in testes of 60 MSG-induced male rats for 180 days. LC was expected to increase the serum levels of testosterone and improve spermatogenesis and stereological characteristics after treatment.

## Materials and methods

### Experimental animals

In this study, 60 adult male Spargue-Dawley rats (10 weeks old, 250 ± 20 g) were provided by the animal laboratory center of Shiraz University of Medical Sciences, Shiraz, Iran. One week before the beginning of the experiments, the animals were acclimated to the experimental conditions. The rats were housed in standard cages (five animals per cage) and were exposed to 12/12-h light/dark cycles. The temperature in the cages was 23 ± 2 °C. The study was approved by the Institutional Animal Ethics Committee. Besides, the experiments were conducted in accordance with the Animal Research: Reporting of in Vivo Experiments (ARRIVE) guidelines [24] for the care and use of research animals.

### Experimental design

MSG (monosodium glutamate, G1626) and LC (C0158) were acquired from Sigma. The rats were randomly divided into six groups of ten, as follows:

1. Healthy control group,
2. Sham group (given 1 mL normal saline as MSG and LC solvent),
3. LC 200 mg/kg b.w. group (given LC 200 mg/kg b.w.),
4. MSG group (given MSG 3 g/kg b.w.),
5. MSG and LC 100 mg/kg b.w. group (administrated with 3 g/kg b.w. of MSG and 100 mg/kg b.w. of LC),
6. MSG and LC 200 mg/kg b.w. group (given MSG 3 g/kg b.w. and LC 200 mg/kg b.w.).

Every morning, 1 mL of treatment was administered by oral gavage for six months; This period was chosen, because rats’ sexual cycle is 60 days and sperms become mature in the epididymis for 12 days [25]. This study was performed on three sexual cycles. We took into account the sham group, which received 1 mL of normal saline (solvent of MSG, LC), in order to evaluate, reduce, and normalized the stress that the oral gavage of rats had caused during our treatment (6 months). A previous research indicated that the ingestion of 3 g/kg b.w. MSG caused reproductive defects in rats. Therefore, the same dose was employed in the present study [26, 27]. Additionally, based on our previous study, the rats were administered with 100 and 200 mg/kg b.w. of LC to evaluate its protective effect on renal damages induced by MSG [28]. At the end of the study, the rats were anaesthetized with a ketamine (10%)/xylazine (2%) mixture (80/5 mg/kg) (Alfasan, Netherlands). Then, 5 mL blood samples were drawn and centrifuged at 3500 rpm for 10 min to separate the serums, which were then kept at -70 °C for further evaluation of biochemical parameters. Finally, the testis tissues were removed for further analyses.

### Determination of serum biochemical parameters

Serum Malondialdehyde (MDA) (nmol/ml), as an indicator of lipid peroxidation [29], was quantified by a thiobarbiturate reactive substance method, as previously described [30]. The Total Anti-oxidant Capacity (TAC) (IU/ml) was measured by the Ferric Reducing Antioxidant Power (FRAP) assay. This method is based on the ability of biological antioxidants in reducing Fe^3+^ to Fe^2+^ in the presence of 2,4,6 tripyridyl-s-triazine (TPTZ). Interaction of Fe^2+^ with 2,4,6-tri(2-pyridyl)- 1,3,5-triazine provides a blue color complex with maximum absorbance at 593 nm [31]. Additionally, the serum levels of LH, FSH, and testosterone were measured by using rat-specific Enzyme-Linked Immunosorbent Assay (ELISA) kits manufactured at the Bioassay Technology Laboratory in Shanghai, China. The samples and standards were incubated in a 96-well plate coated with antibody according to the manufacturer's instructions [32].

### RNA isolation and quantitative RT-PCR gene expression levels

After sacrificing the rats, testis tissues were soaked in an RNA later solution (Qiagen, Hilden, Germany) for 24 h and were then maintained at − 80 °C. Total RNAs were extracted using the Biozol isolation reagent, as recommended by the manufacturer (bioWORLD, OH, USA). The QuantiTect Reverse Transcription Kit (Qiagen, Hilden, Germany) was used to make cDNA from one microgram of RNA in a final volume of 20 μl. The cDNAs were conserved for use in quantitative Real-Time Polymerase Chain Reaction (RT-PCR) experiments. According to a prior study, the ABI 7500 equipment (Applied Biosystems Inc., Foster City, CA, USA) was utilized to assess the mRNA expressions of *Star, Cyp11a1, and Hsd17b3* in the testes [33]. The primer sequences for quantitative RT-PCR analysis have been presented in Table 1. Furthermore, the melting curve was analyzed to evaluate the product specificity, with the slope of the standard curve representing the efficiency of the amplification. Finally, the 2^−ΔΔCt^ method was used to determine relative expression, with the housekeeping gene Glyceraldehyde 3-phosphate dehydrogenase (*GAPDH*) acting as the internal reference [34].

**Table 1:** Forward and reverse primer sequences for particular genes

Primer Direction		Sequences (5′ → 3′)	Length
Forward	***Star* (NM_031558.3)**	AGATGAAGTGCTAAGTAAG	**150**
Reverse		TTGATTTCCTTGACATTTG	
Forward	***Cyp11a1* (NM_017286.3)**	AAAGTATCCGTGATGTGGG	**112**
Reverse		TTTCTGGGCATAGTTGAGC	
Forward	***Hsd17b3* (NM_054007.1)**	ATTACCTCCGTAGTCAAGA	**176**
Reverse		TATTCCACATTCAAAGCCT	
Forward	***GAPDH* (NM_017008.4)**	AAGAAGGTGGTGAAGCAGGCATC	**112**
Reverse		CGAAGGTGGAAGAGTGGGAGTTG	

### Stereological study

**Fig. 1:**
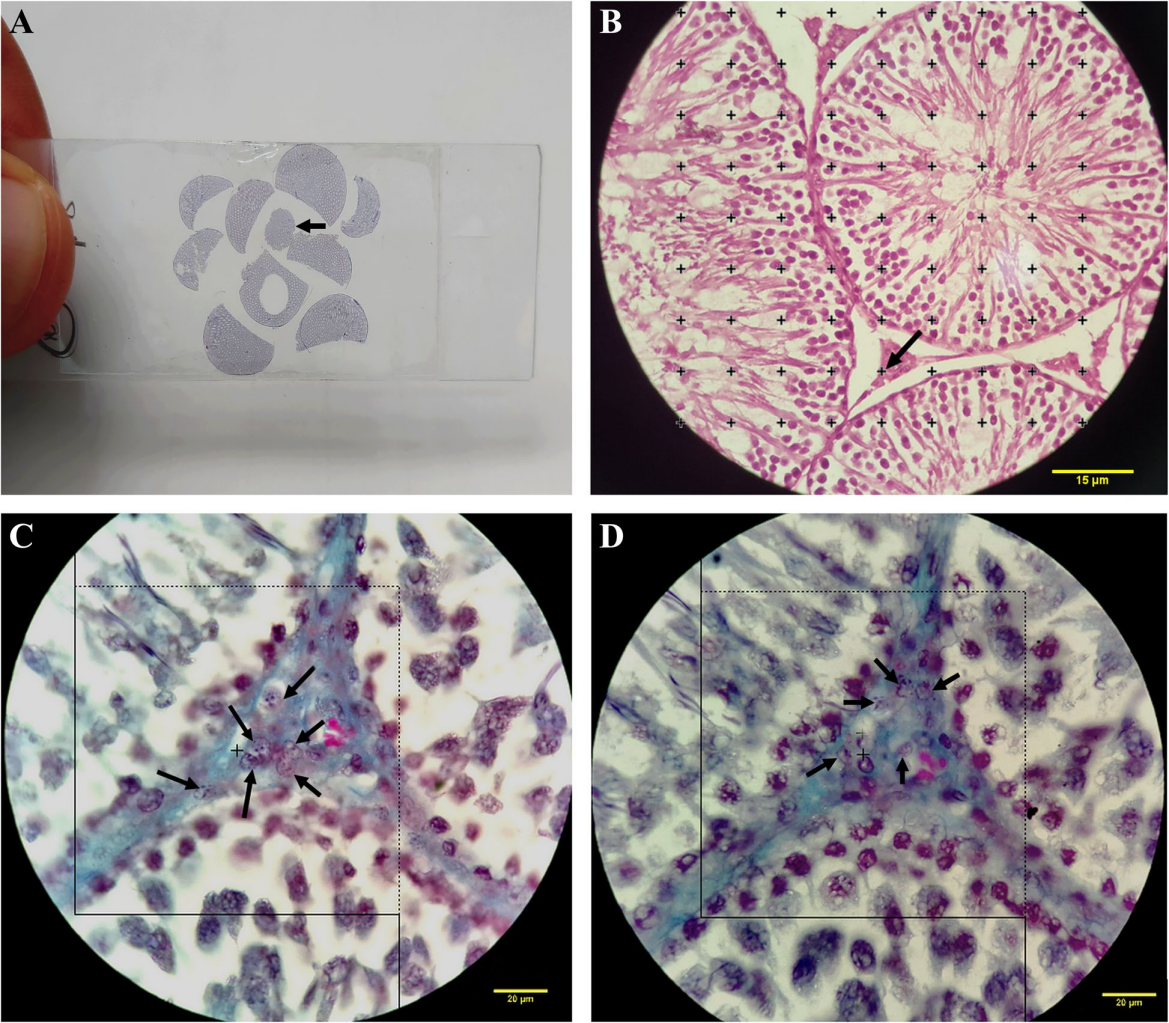
**A** A microscopic slide used to measure shrinkage after staining, with an arrow pointing to a circular portion. **B** Point-counting method; approved points have been displayed by an arrow hitting the right upper corner of each cross of the targeted islets. **C**, **D** Optical disector method; the cells located inside or on the dotted lines and not the solid lines were counted (bottom and left borders)

The testicles were placed in an isotonic saline-filled jar to determine their weight and primary volume [35]. After that, they were fixed in a 10% formaldehyde buffered solution. The orientator method was then used to slice each testis into 8–12 slabs, providing Isotropic Uniform Random (IUR) sections. In doing so, a trocar was used to press a circle out of a testis slab (Fig. 1). The round pieces and all the gathered slabs were placed in a paraffin block. Afterwards, 5 μm and 20 μm thick sections of the paraffin-embedded tissue sections were utilized for volume assessment of the selected items and measurement of cell numbers, respectively. Hematoxylin–eosin staining was used on the 20 μm sections and Masson's trichrome staining was utilized on the 5 μm slices per an established procedure [36]. As previously reported, a formula was used to calculate the overall (post embedding) volume of the whole testis as well as the degree of global tissue shrinkage [d (shr)] [37, 38].

$$Degree\;of\;shrinkage=1-({\frac{AA}{AB})}^{1.5}$$

where *AA* was the area of the circular piece after handling and staining and *AB* was the area of the circular piece before handling and staining.

### Estimating the volumes of the interstitial tissue and the seminiferous tubule

On 5 μm-thick sections, the volume density of the targeted structure (interstitial tissue and seminiferous tubule) was evaluated using the point-counting method (Fig. 1B) and Delesse's formula.

$$Vv\left(structure\right)=\sum_{i=1}^{n}p \left(structure\right)/\sum_{i=1}^{n}(reference)$$

where “${\sum }_{i=1}^{n}p(structure$)” was the number of test points falling on the interstitial tissue and the seminiferous tubule and “${\sum }_{i=1}^{n}p$” was the total points affecting the testis Sects. [39]. The absolute intended structural volume was calculated using the following formula.

$$V\left(structure\right)=V(testis)\times Vv(structure)$$

### Estimating the length, diameter, and height of the germinal epithelium of the seminiferous tubules

The length density of the seminiferous tubules was assessed by randomly placing a fair frame on the screen and quantifying the number at a final magnification of 180 × . The tubule's length density (*LV*) was measured via the following formula:

$$LV=\frac{2\times \sum_{i=1}^{n}Q}{(a/frame)\times \sum F}$$

Where *∑Q* was the total number of tubule profiles determined per rat testis, *a/frame* was the area of the frame used to measure the number, and ∑*F* was the total number of frames measured in each animal. The overall length of the tubule (L) was calculated by multiplying the tubule’s total volume by its length density (LV) [39].

According to Mehranjani et al., in order to estimate the diameter of seminiferous tubules, around 110 to 130 tubules were analyzed. In the counting frame used to estimate the length of the seminiferous tubules, the diameter of the tubules was determined on the sampling tubules. The diameter of the tubules was measured perpendicular to their long axis at the points where they were the widest [40].

The systematic random sample approach was used to assess averagely 8–10 fields from all the 5-m-thick slices of the rats’ testes to quantify the height of the germinal epithelium. This was done using the following equation:

$$H=\frac{Vv}{Sv}$$

where *Vv* was the germinal epithelium's volume density and *Sv* was its surface density.

### Evaluation of the number of sexual lineage cells

The optical dissector method was used to determine the total number of cells including spermatogonia, spermatocytes, round spermatids, long spermatids, Sertoli cells, and Leydig cells in 20 µm sections (Fig. 1 C and D). The total number of the cells was evaluated based on the following formula:

$$Nv=\frac{\sum_{i=1}^{n}Q}{\sum_{i=1}^{n}P\times h\times (\frac{a}{f})}\times \frac{t}{BA}$$

where *∑Q* was the total number of sexual lineage cells observed in all disectors, *h* was the optical disector's height, *a/f* was the area of the counting frame, *∑P* was the total number of observed frames, *BA* was the setting of the microtome, and *t* was the mean of the final section thickness [36]. To estimate the total number of the sexual lineage cells, the following formula was applied:

$${\mathrm N}_{(\mathrm{sexual}\;\mathrm{lineage}\;\mathrm{cells})}={\mathrm N}_{\mathrm v(\mathrm{sexual}\;\mathrm{lineage}\;\mathrm{cells})}\times{\mathrm V}_{(\mathrm{seminiferous}\;\mathrm{tubule}/\mathrm{interstitial}\;\mathrm{tissue})}$$

### Sperm analysis

Epididymal sperms were extracted from the cut edge of the caudal epididymis and were placed in a watch glass filled with 5 mL normal saline solution [41, 42]. After leaving the spermatozoa to diffuse into the solution, the suspension was gently agitated at 37 °C for five minutes to equally distribute the spermatozoa.

#### Sperm motility

The sperm suspension was put on a slide that had been warmed to 37 °C and was analyzed in ten microscopic fields per slide. The fields were chosen randomly for each rat to be examined. Sperm motility was divided into four categories; i.e., quick progressive (spermatozoa moved quickly in a linear way), slow progressive (spermatozoa moved slowly in a linear way), non-progressive (spermatozoa showed circular movements), and immotile (spermatozoa had no circular and linear movements).

#### Sperm count

A Neubauer hemocytometer (Deep 0.1 mm, LABART, Germany) was used to count the sperms under a light microscope at 40 × magnification. The number of sperms was counted in each of the Neubauer chamber's four squares. Then, the total number of sperm cells per mL of semen was calculated by multiplying the mean by 10^6^. It should be noted that the sperms were counted in their entirety (heads and tails included).

#### Estimation of the percentage of abnormal sperms

The suspension was loaded on a slide, stained with 1% eosin Y for 5–10 min, and left to dry. Totally, 200–300 spermatozoa were counted per rat in each sample and the percentage of abnormal sperms was measured. Amorphous heads, two heads, merged body, two tails, and a regularly formed head with a broken or twisted tail were all considered abnormal.

#### Sperm viability

The viability of the sperms was determined by eosin-nigrosin staining. Eosin (Merck, Darmstadt, Germany) and nigrosin (Merck, Darmstadt, Germany) were produced in distilled water. Two volumes of 1% eosin were combined with one volume of sperm suspension. After 30 s, an equivalent volume of nigrosin was added to this mixture. Thin smears were then made and examined at 40 × magnification using a light microscope (Nikon E-200, Japan). In this way, non-viable sperms became red, while viable ones remained colorless.

### Statistical analysis

All statistical analyses were done using the SPSS software (version 23; SPSS Ins, Chicago, USA). The Kolmogorov–Smirnov test was initially employed to assess the normality of the data. Since the data were normally distributed and the variances were homogeneous, parametric tests were utilized. The significance of differences between the means of the variables in the six study groups was determined using one-way ANOVA followed by Tukey's post-hoc test. Statistical significance was defined as *p* < 0.05.

## Results

### L-carnitine improved serum antioxidant capacity in MSG- induced rats

As depicted in Table 2, the level of MDA significantly increased in the MSG group in comparison to the control, sham, and LC 200 groups (*p* < 0.05). However, the MSG groups that received LC 100 and LC 200 as the treatment showed a significant drop in the level of MDA (*p* < 0.05), such a way that there was no significant difference between the MSG + LC 200 group and the control group. According to the results, the level of TAC showed a significant decrease in the MSG group compared to the control group (*p* < 0.05). In the MSG + LC 200 group, LC significantly increased the level of TAC in comparison with the MSG group (*p* < 0.05). Moreover, treatment with LC 100 elevated the level of TAC compared to the control group, but the increment was not statistically significant (Table 2).

**Table 2:** Evaluation of the serum levels of malondialdehyde, total anti-oxidant capacity, LH, FSH, and testosterone in different experimental groups after 180 days of treatment

**Groups**	**MDA (nmol/ml)**	**TAC (IU/ml)**	**LH (IU/ml)**	**FSH (IU/ml)**	**TES (nmol/L) **
**Control**	0.44±0.01	0.33±0.05	22.95±3.27	24.28±3.98	95.28±4.24
**Sham**	0.42±0.01	0.32±0.03	23.45±2.62	26.12±2.53	95.4±8.26
**LC 200**	0.03±0.02	0.34±0.05	23.12±3.67	26.45±2.71	97.31±2.92
**MSG**	1.12±0.04^***^	0.10±0.05^***^	10.33±2.58^***^	12.00±6.41^***^	38.24±4.34^***^
**MSG+LC 100**	0.50±0.31^†††^	0.18±0.04^***^	14.66±1.86^***^	15.00±5.93^***^	52.08±6.63^***^^†^
**MSG+LC 200**	0.24±0.12^†††$^	0.29±0.05^***$$^	18.83±1.16^†††^	20.83±6.89^†^	71.66±11.27^***†††$$$^

Data are presented as mean±SEM, *n*=10

*Compare to sham group; † MSG compare to MSG+LC100 and MSG+LC200 groups; $ MSG+LC100 compare to MSG+LC200. *P*<0.05 considered as significant changes

*MDA* malondialdehyde, *TAC* total anti-oxidant capacity, *TES* testosterone, *LH* luteinizing hormone, *FSH* follicle-stimulating hormone

### L-carnitine increased serum level of LH, FSH, and testosterone in MSG- induced rats

Based on the results presented in Table 2, the serum levels of LH, FSH, and testosterone significantly dropped in the MSG group in comparison to the control, sham, and LC 200 groups (*p* < 0.05). However, there was no significant difference between the MSG + LC 100 and MSG groups regarding the serum levels of LH and FSH. In the MSG + LC 200 group, on the other hand, the levels of LH and FSH increased significantly compared to the MSG group (*p* < 0.05). In both MSG + LC 100 and MSG + LC 200 groups, the level of testosterone significantly rose in comparison to the MSG group (*p* < 0.05).

### Effects of L-carnitine on the mRNA expression levels of Star, Cyp11a1, and Hsd17b3 genes

**Fig. 2:**
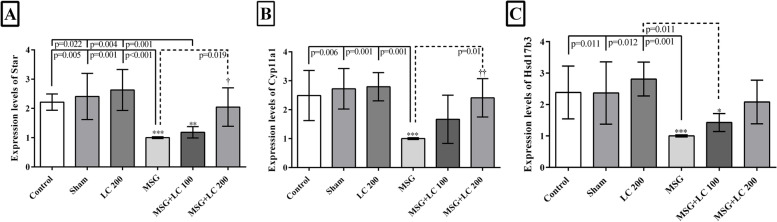
The mRNA expressions of (**A**) *Star*, (**B**) *Cyp11a1*, and (**C**) *Hsd17b3* in different experimental groups of rats after 180 days of treatment. Data have been presented as mean ± SEM. *Compare to sham group; † MSG compare to MSG + LC100 and MSG + LC200 groups; $ MSG + LC100 compare to MSG + LC200. *P* < 0.05 considered as significant changes

According to Fig. 2, compared with the control, sham, and LC 200 groups, the mRNA expressions of all the three genes (*Star, Cyp11a1, and Hsd17b3*) significantly declined in the MSG group (*p* < 0.05). Although the results indicated an increment in the expression levels of *Star, Cyp11a1*, and *Hsd17b3* in the MSG + LC 100 group compared to the MSG group, the differences were not statistically significant. Treatment with LC in the MSG + LC 200 group significantly raised the mRNA expressions of *Star* and *Cyp11a1* in comparison with the MSG group (*p* = 0.019 and *p* = 0.01, respectively), while increase in the mRNA expression of *Hsd17b3* was not significant.

### Stereological study

#### Effects of L-carnitine on the length and diameter of the seminiferous tubules and height of the germinal epithelium

**Fig. 3:**
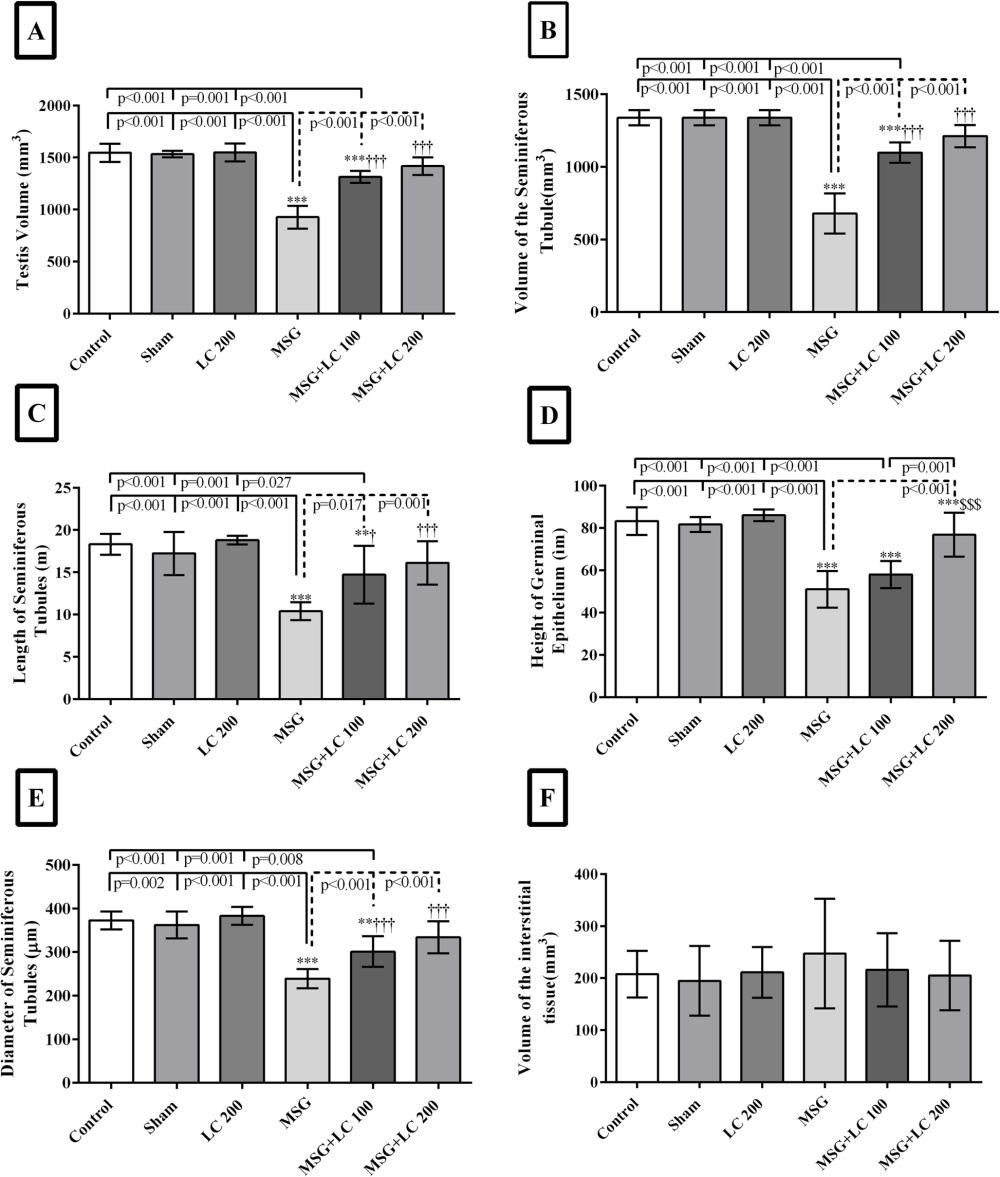
Evaluation of the **A** total volume of the testis, **B** volume of seminiferous tubules, **C** length, **D** germinal epithelium height, **E** diameter of the seminiferous tubules, and **F** interstitial tissues in different experimental groups of rats after 180 days of treatment. *Compare to sham group; † MSG compare to MSG + LC100 and MSG + LC200 groups; $ MSG + LC100 compare to MSG + LC200. *P* < 0.05 considered as significant changes

**Fig. 4:**
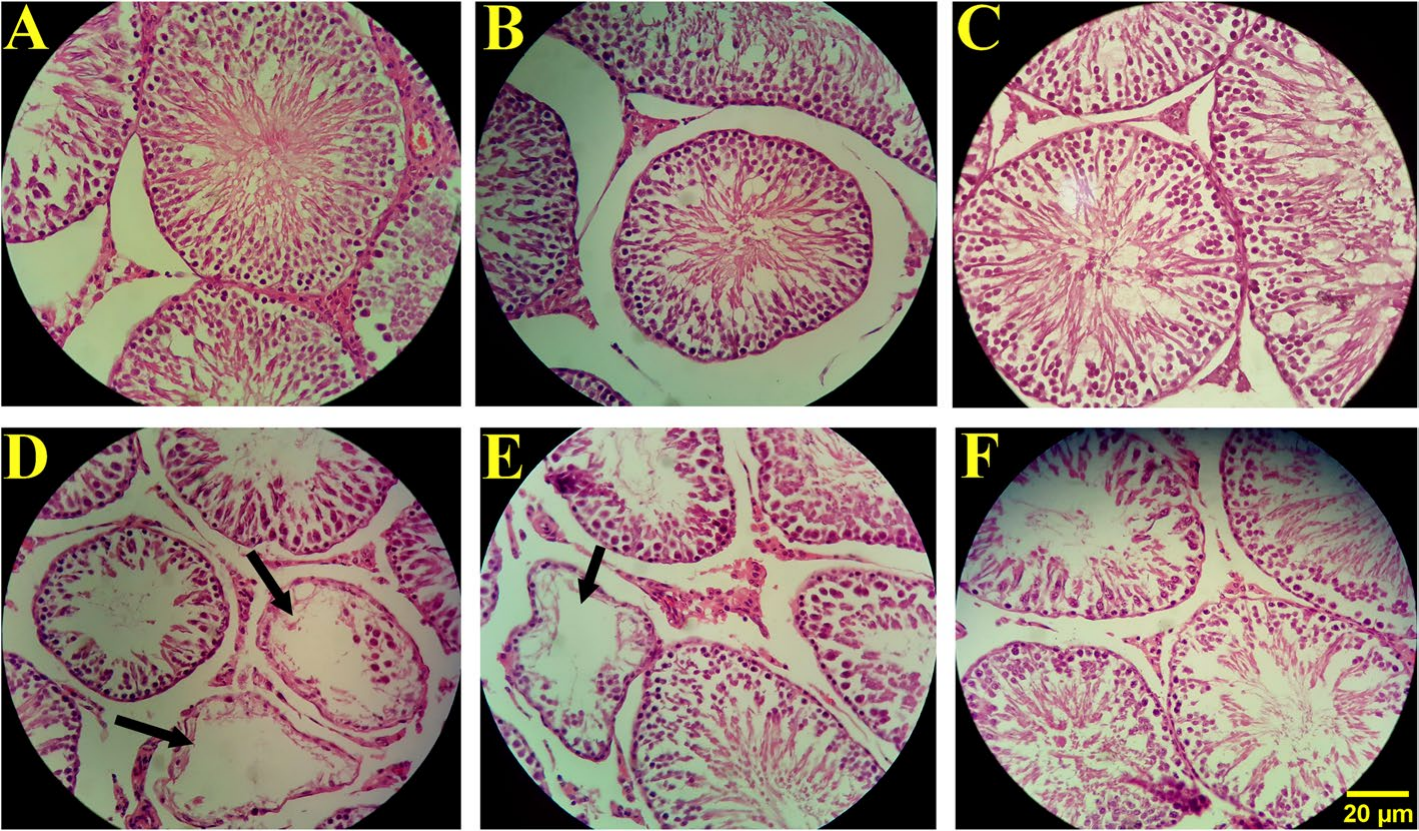
The effect of L-carnitine on testicle tissues and spermatogenesis in MSG-induced male rats (*n* = 10). Control (**A**), sham (**B**), and LC 200 (**C**) groups displayed no histopathological changes, while the MSG group (D) demonstrated degenerative changes in the testis, a reduction in the germinal epithelium height, and loss of sexual lineage cells. In comparison to the MSG group, the MSG + LC 100 group (**E**) exhibited no significant changes. However, the MSG + LC 200 group (**F**) showed a significant improvement in MSG-induced structural impairments such as the loss of sexual lineage cells and deformity in the seminiferous tubules in the testis tissue. The seminiferous tubules seemed atrophic, as shown by the thick arrow (hematoxylin and eosin staining at 40 × magnification)

The results indicated a significant reduction in the total length and diameter of the seminiferous tubules in the MSG group in comparison to the control, sham, and LC 200 groups (*p* < 0.001). Nonetheless, the decline in the total length and diameter of the seminiferous tubules was significantly promoted in the MSG + LC 100 and MSG + LC 200 groups (*p* < 0.05 and *p* < 0.001, respectively). A significant diminution was also found in the height of the germinal epithelium in the MSG group compared to the control, sham, and LC 200 groups (*p* < 0.001). In comparison to the MSG group, LC treatment in the MSG + LC 200 group led to a significant increase in the height of the germinal epithelium (*p* < 0.001), while LC 100 treatment in the MSG + LC 100 group did not make a significant difference (Figs. 3 and 4).

#### Effects of L-carnitine on the total volumes of the testis, seminiferous tubules, and interstitial tissue

The total volume of the testis and seminiferous tubules significantly decreased in the MSG group in comparison to the control, sham, and LC 200 groups (*p* < 0.001). However, in both MSG + LC 100 and MSG + LC 200 groups, the volumes of the testes and seminiferous tubules significantly increased compared to the MSG group (*p* < 0.001). The results revealed no significant difference among the study groups in terms of the volume of the interstitial tissue (Fig. 4).

#### Effects of L-carnitine on the number of sexual lineage cells

**Fig. 5:**
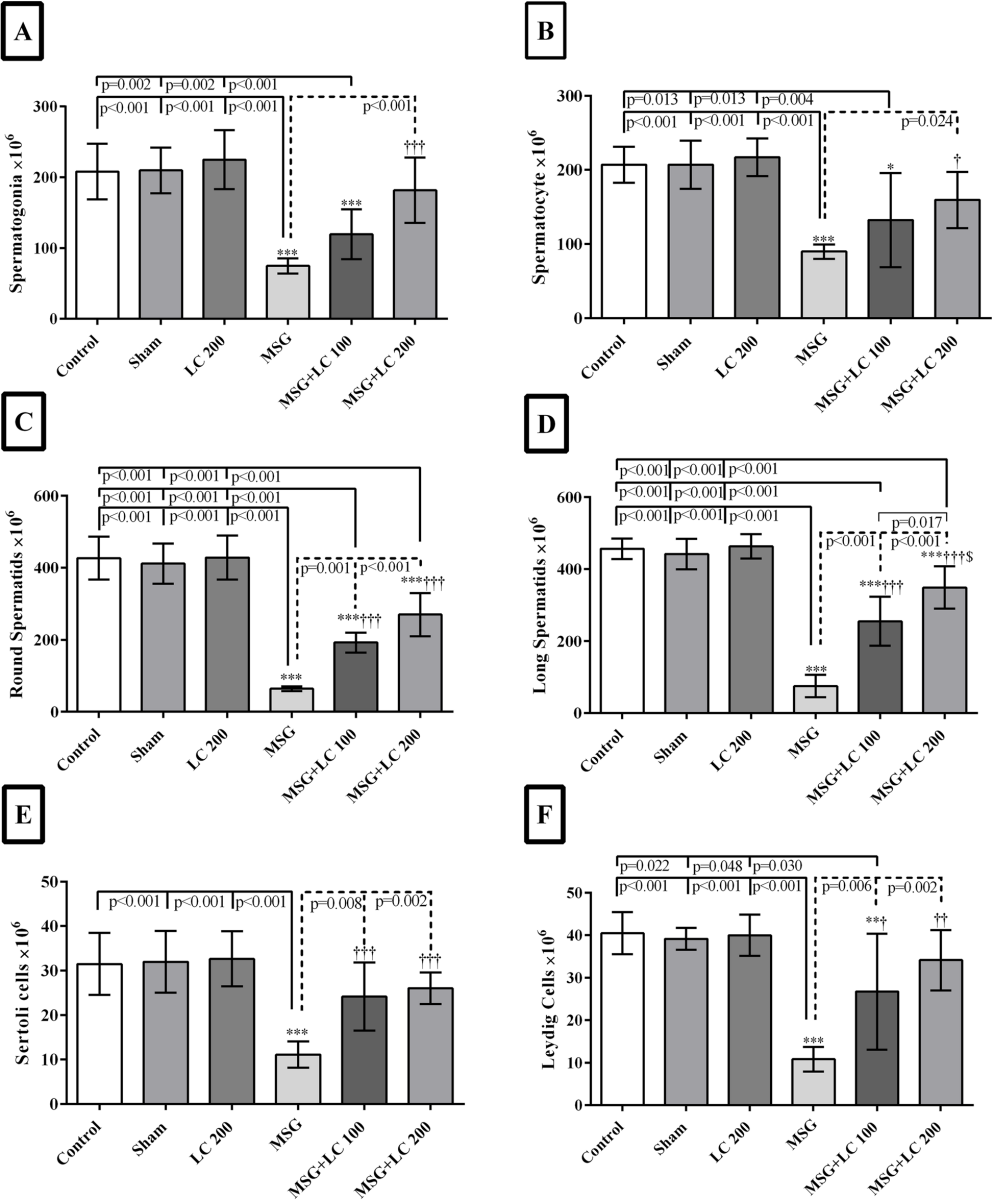
Evaluation of the number of **A** spermatogonia, **B** spermatocytes, **C** round spermatids, **D** long spermatids, **E** Sertoli cells, and **F** Leydig cells in different experimental groups of rats after 180 days of treatment. *Compare to sham group; † MSG compare to MSG + LC100 and MSG + LC200 groups; $ MSG + LC100 compare to MSG + LC200. *P* < 0.05 considered as significant changes

In comparison to the control, sham, and LC 200 groups, the number of spermatogonia, spermatocytes, spermatids (round and long), Sertoli cells, and Leydig cells reduced significantly in the MSG group (*p* < 0.001) (Figs. 4A, B, C, and D, 5). In the MSG + LC 100 group, treatment with LC prevented the loss of Sertoli cells and resulted in a rise in all sexual lineage cells (*p* < 0.01), except for spermatogonia and spermatocytes, in comparison with the MSG group (Figs. 4E, 5). LC also prevented the reduction of Sertoli cells in the MSG + LC 200 group and led to a significant increase in the number of spermatogonia, spermatocytes, round spermatids, long spermatids, and Leydig cells compared to the MSG group (*p* < 0.05) (Figs. 4F, 5).

### L-carnitine improved sperm parameters

The results demonstrated a significant decline in the percentage of rapid progressive sperms (*p* < 0.05), a significant rise in the percentage of non-progressive and immotile sperms (*p* < 0.05), and no significant difference in the percentage of slow progressive sperms in the MSG group in comparison to the control, sham, and LC 200 groups. Compared to the MSG group, treatment with LC 100 and LC 200 significantly increased the percentage of rapid progressive sperms (*p* < 0.05) and reduced the percentage of immotile sperms (*p* < 0.05). Furthermore, the treatment groups were significantly different from the control and sham groups regarding the percentage of rapid progressive sperms and immotile sperms (*p* < 0.05). However, no significant difference was detected between the MSG group and the treatment groups (MSG + LC 100 and MSG + LC 200) with regard to the percentage of non-progressive sperms (Table 3).

**Table 3:** Evaluation of sperm motility in different experimental groups of rats after 180 days of treatment

**Sperm parameters (Mean ± SD)**				
**Groups**	Rapid progressive sperms %	Slow progressive sperms %	Non-progressive sperms %	Immotile sperms %
**Control**	81.19±4.96	7.25±2.19	7.19±2.40	4.35±2.33
**Sham**	77.86±3.17	7.54±2.02	7.63±2.54	6.96±2.25
**LC 200**	81.69±1.48	6.91±1.57	6.86±1.34	4.52±1.91
**MSG**	6.28±1.81^***^	9.14±1.84	21.80±5.47^***^	62.76±3.79^***^
**MSG + LC 100**	20.23±8.34^***^^†††^^$$$^	19.34±1.76^***$$^	15.99±5.29^**^	44.42±13.05^***^^†††^^$$$^
**MSG + LC 200**	45.53±7.78^***^^†††^	14.29±2.67^***^	16.82±3.56^**^	23.34±6.16^***^^†††^

Data are presented as mean±SD, *n*=10

*Compare to sham group; † MSG compare to MSG+LC100 and MSG+LC200 groups; $ MSG+LC100 compare to MSG+LC200. *P*<0.05 considered as significant changes

In comparison to the control, sham, and LC 200 groups, there was a significant drop in the sperm count and a significant increase in the percentage of abnormal morphology and non-viable sperms in the MSG group (*p* < 0.05). Treatment with LC in the MSG + LC 100 and MSG + LC 200 groups significantly increased the sperm count and diminished the percentage of abnormal morphology and non-viable sperms compared to the MSG group (*p* < 0.05). Moreover, significant changes were detected in the sperm count and the percentage of abnormal morphology and non-viable sperms in the treatment groups compared to the control and sham groups, indicating that LC improved the sperm parameters partially (*p* < 0.05) (Table 4).

**Table 4:** Evaluation of the sperm parameters in different experimental groups of rats after 180 days of treatment

**Groups**	**Sperms count ×10**^**6**^	**Abnormal morphology sperms (%)**	**Non-viable sperms (%)**
**Control**	12.97±0.95	7.00±1.54	6.50±1.64
**Sham**	13.23±0.92	6.83±0.98	6.66±1.63
**LC 200**	14.83±0.73	6.16±1.60	4.00±1.41
**MSG**	4.07±0.74^***^	34.66±3.61^***^	65.83±11.51^***^
**MSG + LC 100**	6.07±1.49^***^^†^	15.50±3.27^***^^†††^	45.17±11.75^***^^†††^^$$$^
**MSG + LC 200**	8.96±1.52^***^^†††^^$$$^	13.00±2.36^**^^†††^	24.33±7.68 ^**^^†††^

*Compare to sham group; † MSG compare to MSG+LC100 and MSG+LC200 groups; $ MSG+LC100 compare to MSG+LC200. *P*<0.05 considered as significant changes

## Discussion

MSG is widely used as a food additive in the food industry, but generally without labelling [43, 44]. To the best of our knowledge, this study was the first attempt to investigate the protective effect of LC consumption on sex hormones, mRNA expressions of genes involved in the testosterone synthesis pathway (*Star, Cyp11a1,* and *Hsd17b3*), spermatogenesis, stereological characteristics, and sperm parameters of the testicular tissue in MSG-induced male rats. The study results indicated a significant increment in the MDA concentration and a significant decrease in the TAC level in the MSG group compared to the control and sham groups. Our results were in line with other studies in which treatment of the rats with MSG (4 mg/kg bw) for 28 days [45] and MSG (60 mg/kg BW) for 30 days [46] led to a significant increment in the MDA level. In general, MSG induces oxidative stress by producing free radicals, which causes the pathophysiology of many disorders [28, 47]. Oxidative stress gives rise to testicular tissue damages via DNA damage and increased lipid peroxidation in the long-chain polyunsaturated fatty acids of the testicle tissue [48]. Nevertheless, LC is able to prevent lipid peroxidation by its antioxidant properties [28], which is in accordance with the results of the present investigation. In our study, the MSG groups receiving LC (100 and 200 mg/kg b.w.) as an antioxidant supplement showed a significant drop in the MDA levels and an increase in the TAC levels compared to the MSG group. The other studies showed similar effects of LC on raising the level of TAC in the adult Wistar rats [49] and a boost in the MDA level of the experimental testicular torsion-detorsion model of rats [50].

According to the current study findings, the serum levels of LH, FSH, and testosterone significantly decreased in the MSG group in comparison to the control and sham groups. However, the levels of LH, FSH, and testosterone significantly increased in both MSG + LC 100 and MSG + LC 200 groups in comparison to the MSG group. Various studies have shown decreased levels of LH, FSH, and testosterone following MSG administration. The main reason for this decline may be the impact of MSG on the hypothalamic-pituitary–gonadal axis. It has been proven that MSG causes Central Nervous System (CNS) nerve cell destruction in hypothalamus and pituitary gland, which can affect Gonadotropin-Releasing Hormone (GnRH) secretion [51–54]. In other words, nerve cells destruction has a detrimental impact on the cells producing LH and FSH in the anterior pituitary gland, which eventually results in a decline in the serum levels of LH and FSH. Furthermore, if MSG effects occur on hypothalamus and GnRH-producing cells, the serum level of GnRH will be remarkably reduced. The decrease in GnRH can, in turn, reduce LH and FSH hormones. A limitation of the current study is the absence of measurement of serum GnRH levels and hypothalamic-pituitary axis, which should be addressed in follow-up research.

At the molecular level, the mRNA expressions of all the three genes (*Star, Cyp11a1, and Hsd17b3*) significantly dropped in the MSG group compared with the control and sham groups. *Star*, *Cyp11a1,* and *Hsd17b3* genes play a vital role in the process of converting cholesterol to testosterone. *Star* transports cholesterol to the inner membrane of the mitochondria. *Cyp11a1* then converts the cholesterol molecule to pregnenolone in the inner membrane of the mitochondria. Afterwards, *Hsd17b3* catalyzes the formation of testosterone from androstenedione [33]. The reduction in the expression of these three genes led to a drop in the level of testosterone in the serum, according to the current study's molecular findings, which confirmed the hormonal findings. The study results revealed that treatment with LC in the MSG + LC 200 group significantly raised the mRNA expressions of *Star* and *Cyp11a1* in comparison to the MSG group. Therefore, high doses of LC could result in a significant boost in the serum level of testosterone compared to the MSG group. According to an investigation, treatment with LC (500 mg/kg) on carbendazim-challenged rats resulted in overexpression of *Star* mRNA, which was in line with our study because carbendazim also generates reproductive toxicity in the male rats [55]. The exact molecular mechanism of L-carnitine on the testosterone synthesis pathway have remained unknown and required further research.

Histopathological findings revealed a significant decrease in the testicular volume, length and diameter of the seminiferous tubules, germinal epithelium height, motility, and viability in the MSG group compared to the control and sham groups, which were consistent with the findings of other reports [54, 56, 57]. However, the reduction of the mentioned parameters was significantly prevented in the group receiving LC 200 mg/kg. The investigations have shown that MSG has toxic effects on the testicular tissue, causing oligospermia and increasing abnormalities in sperm shape and morphology in Wistar rats [58, 59]. It is also emphasized that MSG consumption causes infertility in males via hemorrhage, degeneration, and changes in sperm population and morphology [52]. MSG also leads to cell apoptosis and necrosis by producing free radicals and breaking down DNA molecules. Moreover, MSG inhibits the production of germ cells, subsequently reducing the number of spermatogonia, spermatocytes, and spermatids and leading to oligospermia in the testicular tissue by affecting DNA molecules [60]. Therefore, reduction and disappearance of the germ cell lineage will be accompanied by a decline in the volume of testes, diameter and length of seminiferous tubules, and height of the germinal epithelium.

The present study indicated that in comparison to the control group, the number of spermatogonia, spermatocytes, spermatids (round and long), Sertoli cells, and Leydig cells reduced significantly in the MSG group. An earlier discovery indicated that FSH is required to induce spermatogenesis; hence, a reduction in FSH lowers the testicular volume and stereological parameters [61]. Along with FSH, LH contributes to the secretion of testosterone from Leydig cells and testosterone spermatogenesis [6]. A decline in the level of LH can result in a decrease in the level of testosterone, thereby reducing spermatogenesis. As the process of spermatogenesis declines, the volume of the testis, height of the germinal epithelium, number of sexual lineage cells, and percentage of normal and viable sperms witness a reduction, as well.

Through the following pathways, LC appears to improve the state of sexual cells. First off, because of its antioxidant characteristics, it can be quite effective in defending the membranes of sexual cells against free radicals and oxidative stress [62]. In fact, LC protects against ROS-related harm to DNA and cell membranes. Second, sperm cells' mitochondrial long-chain fatty acid metabolism is impacted by LC [63]. Before passing through the mitochondrial membrane, fatty acids must first bond to acetyl coenzyme A. It, in turn, requires LC as a cofactor. Hence, LC facilitates lipid metabolism and produces energy for sperm motility [64]. It has been shown that the concentration of LC is 2000 times higher in the epididymis and sperm cells than in the plasma [65].

The results of the current investigation revealed that LC administration improved sperm motility, progression, and viability, which was in agreement with the results of other investigations [66–68]. For instance, Kang et al. demonstrated that LC consumption considerably reduced the apoptotic cells located in seminiferous tubes [69]. Lenzi et al. also carried out a research on 86 infertile males and revealed the positive effect of LC on increasing fertility, sperm count, and motility [70]. In another study performed by Vitali et al. on 47 patients, LC induction increased the number of sex cells, motility, and sperm count [71]. Therefore, LC can regulate metabolism and function in Sertoli cells; this molecule directly influences testicular sperm maturation via stimulating glucose uptake by Sertoli cells. In a prior research, adding LC to Sertoli cell culture medium significantly increased the secretion of pyruvate and lactate, which are essential for energy production and maturation of sperm cells in the testis [72]. Moreover, studies demonstrated that the onset of sperm motility occurred consistently with an increase in LC in the epididymal lumen and a rise in acetyl-L-carnitine concentrations in spermatozoa [73, 74]. Accordingly, the accumulation of LC provides sperms with the ability of progressive movement and fertilization. LC also enhances cell viability via the release of cellular enzymes and oxygen consumption [15]. Hence, androgenic storage of free LC and acetyl-L-carnitine in mature and ejaculated sperms is a guarantee for sperm viability [75]. Taken together, MSG as a food additive could lead to reproductive abnormalities on the molecular, biochemical, and histopathological levels in the male rats. However, LC ameliorated the MSG induced testicular toxicity to great extent and enhanced steroidogenesis, spermatogenesis, sperm parameters. LC can improve male reproductive performance and may be its antioxidant effect is the major reason for L-carnitine-mediated tissue protection.

## Conclusion

The study results revealed that LC 200 mg/kg could enhance the TAC level and decrease the MDA level. In addition, LC contributed to an increment in the levels of LH, FSH, and testosterone (sex hormones) and improved cell function via raising the mRNA expression levels of *Star*, *Cyp11a1*, and *Hsd17b3* in the testicle tissues of MSG-induced male rats. LC also increased sperm motility, sperm survival, and reduced sperm morphology abnormality. The findings of the study revealed that L-carnitine, because of its antioxidant characteristics, reduced the defects in rat reproduction brought on by MSG.

## Data Availability

The datasets used and analyzed during the current study are available from the corresponding author on reasonable request.

## Abbreviations

(*Cyp11a1*): Cytochrome P450 family 11 subfamily a polypeptide 1
(ELISA): Enzyme Linked Immunosorbent Assay
(FSH): Follicle-Stimulating Hormone
(GnRH): Gonadotropin-Releasing Hormone
(LC): L-carnitine, (LH): Luteinizing Hormone
(MDA): Malondialdehyde
(MSG): Monosodium Glutamate
(ROS): Reactive Oxygen Species
(*Star*): Steroidogenic Acute Regulatory protein
(TAC): Total Anti-oxidant Capacity
(*Hsd17b3*): 17β-Hydroxysteroid dehydrogenase-3
